# Some Minor Manifestations of Epilepsy

**Published:** 1910-05-28

**Authors:** R. O. Moon


### Hospital Clinics.

### SOME MINOR MANIFESTATIONS OF EPILEPSY.

By R. O. MOON, M.D., F.R.C.P.

(Delivered at the Seamen's Hospital, Greenwich, on Thursday, April 28, 1910.)

Gentlemen,—I thought that we would speak this afternoon not about the classical epileptic fit, but rather about some of those minor manifestations of epilepsy which are not so obvious, and whose importance is not so frequently appreciated. Petit mal is a well-recognised aspect of epilepsy. It is, as you know, a momentary loss of consciousness, with perhaps some slight convulsive movement, which, in many cases, is not much noticed. The patient does not cry out in it, nor fall to the ground, but there may be some drooping of the head, and after the attack he may appear peculiar and may do strange things.

### Examining the Patient.

Attacks of petit mal are, in the ordinary course, easy of recognition. The mother says the child is fairly well, but that it has fits and faints, which are sandwiched in together. But a more puzzling kind of case is this: A child will be brought with the statement that it is losing flesh, has a bad appetite, and is very tired and languid. Perhaps the mother will say that it has fainted two or three times recently. The child will generally be found to be rather a wretched specimen, anæmic and lack-lustre. On examining the chest, you may find a systolic murmur at the apex. There may be a history of rheumatism, and you may not unnaturally conclude that it has got some form of heart disease. Perhaps you prescribe iron and cod-liver oil. There may be improvement, but not long afterwards you see it again, because there has been an epileptic fit. On looking close into the case you find that the mental faculties are not what they should be. This throws light on the fainting attacks, and you realise that the condition is petit mal. I do not think cardiac faints are at all common in children. But here are some points of distinction between syncope and petit mal. In both the patient loses consciousness; but in the cardiac faint the onset is slow. The person who is going to faint does not fall down at once, he has some warning in the shape of increasing faintness; but the petit mal attack is sudden. Recovery from a cardiac faint is slow, but the mind is clear; he is aware of surrounding objects, and his mind is not confused and dazed. The petit mal patient comes to rapidly, but he has a dazed mental feeling and may have headache. In a cardiac faint there may be some obvious cause, such as a hot and close room, over-fatigue, or some emotional excitement; whereas the patient with petit mal will go off without any such obvious reason. The frequency of such attacks must be ascertained by questioning the mother or the nurse. If you saw an attack, and had the hand on the pulse, you would find in the epileptic attack the pulse would not be appreciably altered, whereas in the syncopal attack it would get slower and slower. You will see the importance of the distinction when we come to speak of treatment.

The next manifestation of epilepsy is night terrors. There are different causes for these. Episepsy is a frequent cause, but the cause may be no more remote than the patient having had a late and heavy supper. I think the most common cause is the rheumatic diathesis; rheumatic fever is very common in children, and it affects the heart, but you may not be so familiar with the fact that it damages the nervous system, making the subjects of it extremely neurotic and irritable. The child may go to bed in the ordinary way, apparently quite well; but not long after it has gone to sleep it suddenly awakes shrieking and shouting, and with the eyes staring, gazing, it may be, into a corner of the room, as if it saw there some horrible vision. Having been soothed off to sleep, next morning nothing seems to be known of the occurrence. As to the connection of night terrors with epilepsy, here are two or three notes of cases.

A small girl, æt. 5½ years, was brought to me because of night terrors; she woke up crying at times, and had twitching movements during sleep. In the morning she feels sick. A year later she was going on very well; no longer any night terrors, but there were occasional twitching movements during sleep. Three years later she came because two months previously she had had an attack which was thought to be epilepsy. She had two more in the course of the next few days. There were points in the family history which might have made one suspicious. The mother had epilepsy in childhood, and also suffered from somnambulism. Two maternal aunts were in an asylum, the father was addicted to alcohol, and in one or two cases alcohol in the parents seems to have had some bearing on the child's condition; it seems to assist in producing epilepsy. She had a high-arched palate, and that is a point to which alienists attach considerable importance as associated with epilepsy.

Another case was that of a boy 3½ years of age, who was brought for night terrors, which had occurred every two months. He woke up soon after going to bed imagining that all sorts of things were pursuing him. He is of a nervous disposition, being afraid of the dark and afraid of horses, and he trembles when crossing the road; otherwise the general health was good. A few months ago, at the onset of measles, he had a fit, and since then several more, which were of an ordinary epileptic character. The family history shows that the father had fits as a child, and the maternal grandfather suffered from them; the palate is high-arched. This one may regard as more than ordinary night-terrors.

The next was a girl 13 years of age, suffering from night terrors. On inquiring into her previous history, it appears she had a convulsion while teething, at the age of eight months, and continued to have three or four fits a year. It is reasonable to think there was some connection between the two, as her mother suffered from fits in early life, the father was addicted to alcohol, and she had a high-arched palate.

Another manifestation of epilepsy is nocturnal enuresis, which is very common. There are plenty of causes of it: phimosis, worms, and in girls vulvo-vaginitis, which means that there is local irritation, Some people attach importance to great acidity of the urine, irritating the bladder. More common is the neurotic child, who is healthy, and after a time gets cured. But there are some cases in which the enuresis is something more than this; it is a manifestation of epilepsy. It is not unreasonable that it should be so, because in a classical fit of epilepsy the patient may pass his water. In fact, when you want to know whether a fit is hysterical or not, you ask the patient whether he has bitten his tongue or lips, or passed his water in bed. In the cases where there is enuresis as a sign of epilepsy there is generally some periodicity about it; once a week, once a fortnight, or once a month. To give you evidence of this, I will draw your attention to two or three cases.

A boy, æt. 8 years, suffered from nocturnal enuresis for almost a year, and from night terrors for the last four years. At the age of 2½ he had a definite epileptic fit. When seen a year later the enuresis was still intractable, but he also suffered from sleep-walking and great irritability in the morning. The symptoms are suggestive of epilepsy.

Here is another instance, perhaps more striking. A boy æt. 7. A year previously, when he was 6, he had an epileptic fit, followed by another a month later. Soon after, he began to have nocturnal enuresis, was strange in his manner, irritable, and backward mentally.

A girl, æt. 9, suffered from enuresis for a year, and it resisted all treatment. In 1908 she was in hospital sixteen weeks, but was discharged as incurable. While there she had two fits, and often has outbursts of temper, and her memory is bad. This intractability suggests that the enuresis may be of epileptic origin. I may say that when in hospital the patient did not wet the bed once. Whether that is due to the more regular diet and life there I do not know.

Another instance is that of a boy, æt. 11, who suffered from enuresis from æt. 4; otherwise he is in good bodily health. He had convulsions after influenza, and is mentally backward, irritable, passionate, and destructive. This child should be watched carefully at puberty to see if there is an outbreak of epilepsy. It is a common time for children to have epilepsy, and women are liable to have it after the commencement of the catamenia.

We now pass to more debatable ground; paroxysmal headaches, often known as migraine. Such headaches have often been observed to interchange with epilepsy, and people have thought there is some resemblance between the two; thus a patient has epilepsy in early life, and later loses his epilepsy and has migraine; some people start with migraine in early life, and later develop epilepsy. There are certain superficial resemblances between epilepsy and migraine. Epilepsy is paroxysmal; you get a sudden warning, and then there are the motor phenomena, the tonic and clonic contractions; there is the post-epileptic headache, which is, of course, a sensory phenomenon. Epilepsy is regarded as a discharge of cortical cells. The idea is that there is a great deal of force accumulated in the cortex, and all of a sudden some stimulus sets this force going; hence arises the fit. Migraine is somewhat similar, only it is sensory; it may begin with visual spectra. These can be compared wth the auræ of epilepsy. And then comes the main attack which lasts longer, a severe and painful throbbing headache, which you might regard as the sensory equivalent of the motor phenomena of epilepsy. In migraine there is one motor phenomenon—namely, vomiting. I have got some clinical evidence on the subject. A girl, æt. 13, has definite migraine about every week. Two years previously she had attacks in which she lost consciousness, obviously petit mal attacks. Three or four months went by, but she did not get stronger, and suffered from epileptic fits. Her father was addicted to alcohol.

A girl, æt. 7, often wakes in the morning with a headache, lasting about an hour, and then vomits. At times she was said to lose consciousness, and often talks to herself. What suggests epilepsy is that she had a fit when fifteen months old, and was unconscious fifteen hours. Secondly, she is very irritable and passionate. Her palate is high-arched, and the paternal grandmother suffers from epilepsy. Heredity is a most important element in all cases of epilepsy.

A boy, æt. 6, for the last three years has suffered from paroxysms of headache once weekly. The headache begins in the forenoon, and gradually grows worse towards the evening. Usually he is sick at an early stage of the headache, and says that before being sick everything goes red in front of him. Afterwards he is tired and languid, and there is no headache. In the last two months he has had epileptic fits, and the paroxysmal headache has been most frequent. Often in the evening he had night terrors, which are suggestive of epilepsy. I will explain presently the importance of being able to recognise migraine as possibly a manifestation of epilepsy.

Another manifestation is somnambulism. In some cases there are abdominal symptoms. A girl, æt. 7, once a fortnight has severe pains in the abdomen, of which there is no relief until she vomits, and then she has a deep sleep, which sometimes lasts for hours, with slight twitching during sleep. The attacks usually occur on the same day of the week. The points which suggest epilepsy here are the fact that she has had convulsions in infancy, that one sister has had a fit in childhood, that she suffers from enuresis at night, night terrors, and there is a periodicity in the attacks.

The next condition is epilepsia rotatoria, or rotatory epilepsy, where the patient runs round in a circle. Sometimes epilepsy shows itself purely as the aura of a fit. A child two years old used to have a fit every two months, and prior to that went round in a circle. It is ordinary epilepsy with this peculiar aura, but often he turns round in a circle and falls down, without having a fit.

### Incomplete Forms.

I will now tell you of one or two instances of incomplete forms of epilepsy. I published them a few years ago in the *Lancet* (1904). Two sisters, æt. respectively 5 and 3½, suffered with periodic attacks of shaking of the arms. Soon after they were able to walk they would stop suddenly, look on the ground, begin shaking, especially when they were not occupied. Both children are intelligent, but irritable. The father suffers from epilepsy.

Here is a rather more striking case. A boy, æt. 8½, began six months previously to have attacks, of retching, without being actually sick. In a few minutes he felt all right again. Lately the attacks have changed in character, for he now moves his arms, but does not lose consciousness. He can speak, but his voice is peculiar, and he has a strange look. Prior to the attack he feels as if he were going to be sick, and works his arms so as to prevent it. These attacks last two minutes, and then he feels well again. There had been no definite epileptic fits. Doubtless these phenomena were of epileptic character.

Another manifestation is a wandering impulse, of which the Germans have written much. A child will suddenly leave school, or its home, and disappear for half a day or so and then return, or is found by somebody and does not know what he has been doing.

### Prognosis.

Epilepsy is not dangerous to life, as a rule; occasionally cases are known in which the patient dies of status epilepticus, and, of course, they sometimes die from accident. So that if it were only in these manifestations that epilepsy consisted, it would not be such a very serious condition; but it is in the mental and moral qualities of the patient that the seriousness consists. Subjects of even these minor manifestations you will find, if you follow them up, are not satisfactory mentally and morally. There are deficient memory and attention, also a lack of concentration, these being the chief essentials for education. I do not say that some of these children are not quite clever and intelligent, but it is an ill-balanced cleverness. And you find they are passionate, have outbursts of temper, and are destructive, irritable, and peculiar. To be forewarned is to be forearmed, and realising what the conditions are, you will be in a better position to treat them. But in treating the night-terrors, enuresis, and somnambulism you are only treating the symptoms, whereas you need to attend to the underlying condition.

With regard to treatment, my first counsel is educational; the child should be fully occupied, and so should attend school; though if the child is constantly having these attacks it cannot very well attend school, because of the effect on the other children. But such a child must not be over-worked or over-stimulated, as is not unlikely to happen when the child is clever, as these children very often are, though in an ill-balanced way. Working for exams. is nearly always bad. Then there is the child who is rather stupid and over-conscientious, and will work to the top of its bent and do itself harm in the process. And the child of passionate disposition and peculiar habits is apt to be a good deal baited at school. But it is not so much the amount of instruction imparted which is beneficial as the discipline and regular methods which are introduced into the child's life. The instruction should be physical and manual, rather than intellectual. If the child later on is seriously damaged in mental capacity it will yet have acquired some muscular facility for doing things, and will be able to do a certain amount of work if it has been properly taught.

I have not much to say about diet, the most important point in regard to which is regularity of meals, and moderation in quantity. I reduce meat a good deal, at times omitting it altogether. Tea and coffee may be well omitted from the dietary of children under 12.

With regard to drugs, in all treatment of epilepsy bromides are the sheet anchor; but in the minor manifestations they must be given with a sparing hand. The night terrors are easily controlled by moderate doses of bromide. Enuresis is more troublesome, but belladonna combined with bromide will generally be effective. Somnambulism is generally controlled by bromide. Constipation should be avoided, as it is likely to bring on an attack of grand mal or petit mal. For a child between 6 and 14 years of age I find 20 minims of extract of cascara and 20 minims extract of liquorice, given once or twice a day, a useful prescription for this purpose. Another drug which is useful, particularly in petit mal, was first introduced by Sir William Gowers—namely, borax, or borate of soda, in doses of 5 to 15 grains for a child ten years of age. I do not think it is much good by itself, but is useful in addition to bromide. Large doses of it are apt to give children diarrhœa. There are some cases of epilepsy in children where tonic treatment is useful for a time—namely, cod-liver oil and iron, particularly in cases of minor manifestations.

One has to think of epilepsy not so much as a single disease but as a collection of many diseases; it is an outward and visible sign of a nervous system which is damaged in some way which we do not entirely understand. Therefore, all these small points are important with regard to the prognosis, because your method of advising with regard to the education and training and the whole life of the child will be guided very much by the extent to which you think these manifestations are epileptic. And therefore you should not be content with just getting rid of the symptom, which may not be in itself particularly serious or troublesome, but have regard to the disease because of what it implies and foreshadows.

